# A Role for Matrix Metalloproteases in Antidepressant Efficacy

**DOI:** 10.3389/fnmol.2019.00117

**Published:** 2019-05-08

**Authors:** Seham Alaiyed, Katherine Conant

**Affiliations:** ^1^Department of Pharmacology, Georgetown University Medical Center, Washington, DC, United States; ^2^Department of Neuroscience, Georgetown University Medical Center, Washington, DC, United States

**Keywords:** matrix metalloproteases, perineuronal nets, depression, monoamines, gamma oscillations

## Abstract

Major depressive disorder is a debilitating condition that affects approximately 15% of the United States population. Though the neurophysiological mechanisms that underlie this disorder are not completely understood, both human and rodent studies suggest that excitatory/inhibitory (E/I) balance is reduced with the depressive phenotype. In contrast, antidepressant efficacy in responsive individuals correlates with increased excitatory neurotransmission in select brain regions, suggesting that the restoration of E/I balance may improve mood. Enhanced excitatory transmission can occur through mechanisms including increased dendritic arborization and synapse formation in pyramidal neurons. Reduced activity of inhibitory neurons may also contribute to antidepressant efficacy. Consistent with this possibility, the fast-acting antidepressant ketamine may act by selective inhibition of glutamatergic input to GABA releasing parvalbumin (PV)-expressing interneurons. Recent work has also shown that a negative allosteric modulator of the GABA-A receptor α subunit can improve depression-related behavior. PV-expressing interneurons are thought to represent critical pacemakers for synchronous network events. These neurons also represent the predominant GABAergic neuronal population that is enveloped by the perineuronal net (PNN), a lattice-like structure that is thought to stabilize glutamatergic input to this cell type. Disruption of the PNN reduces PV excitability and increases pyramidal cell excitability. Various antidepressant medications increase the expression of matrix metalloproteinases (MMPs), enzymes that can increase pyramidal cell dendritic arborization and spine formation. MMPs can also cleave PNN proteins to reduce PV neuron-mediated inhibition. The present review will focus on mechanisms that may underlie antidepressant efficacy, with a focus on monoamines as facilitators of increased matrix metalloprotease (MMP) expression and activation. Discussion will include MMP-dependent effects on pyramidal cell structure and function, as well as MMP-dependent effects on PV expressing interneurons. We conclude with discussion of antidepressant use for those at risk for Alzheimer’s disease, and we also highlight areas for further study.

## Introduction

Major depression is a life-threatening disorder that imparts substantial pain and suffering on affected individuals as well as their friends and family members. First-line therapy typically involves the use of specific monoamine reuptake inhibitors. While these drugs are effective in a subset of patients, for other patients they have little to no benefit. An improved understanding of the molecular and neurophysiological mechanisms that contribute to improved mood in responsive individuals could drive the development of novel drugs or drug combinations. The present review is focused on the potential for increased MMP activity to contribute to antidepressant efficacy. We also discuss the possibility that pre-emptive use of monoamine reuptake inhibitors that increase MMP activity could benefit patients who are likely to develop Alzheimer’s disease, a disorder for which untreated major depression is a significant risk factor.

## Major Depression is Linked to Regional Reductions in Brain Volume and Attenuated Glutamatergic Transmission

In a human study, hippocampal volume was reduced in association with depression and increased in association with a therapeutic response to antidepressant therapy (Joshi et al., 2015). Moreover, in a large consortium analysis spanning 200 institutions in 35 countries, human hippocampal volume was reduced with major depression (Thompson P.M. et al., 2015). The magnitude of hippocampal volume reduction has also been linked to disease duration (Kessing and Nilsson, 2003). In a recent meta-analysis, increased hippocampal volume was identified as the most promising marker for disease remission in response to antidepressant treatment (Chi et al., 2015). In rodent models both electroconvulsive therapy and imipramine have been shown to increase hippocampal volume (Chen et al., 2008, 2009, 2010). In contrast, in an unpredictable stress-induced model of depression hippocampal volume is reduced (Schoenfeld et al., 2017).

It has been suggested that anti-depressant therapy might increase hippocampal volume through the generation of new neurons as well as through specific structural changes in dendrites of pre-existing neurons (Chen et al., 2008, 2009, 2010). The latter effect may be of particular importance to treatment efficacy, since increased dendritic branching and formation of new dendritic spines would allow for an increase in the number of post-synaptic contacts for glutamate. Structural analyses suggest that the large majority of inputs to the dendrites of glutamatergic neurons are excitatory (Kasthuri et al., 2015), and functional studies have linked increased dendritic arbor with an increased neuronal calcium response (He et al., 2016).

Glutamatergic transmission is indeed reduced with depression (Kuhn et al., 2016). Various human and rodent studies suggest that depression is linked to reductions in the overall strength of glutamatergic transmission in the hippocampus [reviewed in Thompson S.M. et al. (2015)]. Published work has also demonstrated an association between stress and reduced mRNA for the glutamate receptor subunit GluA1 within the hippocampus, as well as impaired long-term potentiation (LTP) of synaptic transmission (Alfarez et al., 2003; Schmidt et al., 2010). Moreover, autopsy studies have shown that multiple glutamate receptor subunits are reduced in patients that were diagnosed with depression (Choudary et al., 2005; Beneyto et al., 2007).

Agents that enhance monoaminergic transmission can stimulate dendritic branching and spine formation in various neuronal subtypes. For example, one study showed that the number of spines on excitatory neurons was increased in the hippocampus following 14 days of treatment with imipramine as compared to saline (Chen et al., 2008). Imipramine also enhances the complexity of immature neurons in the granule cell layer (Fenton et al., 2015) while methamphetamine and cocaine increase dendritic arbor and spine number in striatum (Robinson and Kolb, 2004). Furthermore, the noradrenergic and serotonergic tetracycline antidepressant mirtazapine can increase dendritic arbor in the sensory cortex of MeCP2 null mice (Bittolo et al., 2016).

Monoamines have also been shown to enhance hippocampal neurogenesis (Jhaveri et al., 2010), though whether newborn neurons influence excitatory/inhibitory balance in a widespread manner is unclear. Work from Duman and colleagues has shown that antidepressant therapies including the selective serotonin reuptake inhibitor (SSRI) fluoxetine can enhance neurogenesis in the adult rat hippocampus, and that this effect requires chronic (14 days) as opposed to acute treatment (Malberg et al., 2000). In terms of therapeutic relevance, neurogenesis in the adult hippocampus is important for the antidepressant fluoxetine to prevent stress-induced anhedonia in rodents (Miller and Hen, 2015), and newborn neurons also reduce the activity of stress-responsive neurons in the ventral dentate gyrus (Anacker et al., 2018).

## Increased Matrix Metalloprotease Activity Might Ameliorate Depression-Related Changes in Brain Structure and Function

### An Introduction to Matrix Metalloproteases

Matrix metalloproteases (MMPs) are zinc-dependent endopeptidases that are typically released as pro-forms from intracellular stores. Extracellular activation is generally achieved through cleavage of the pro-domain which exposes the catalytic region. Pro-domain cleavage is mediated by previously activated MMPs and by additional proteases including plasmin (Baramova et al., 1997; Toth et al., 2003). Cleavage-independent changes in tertiary structure that allow for exposure of the catalytic domain may also follow from nitrosylation and alternative oxidation- dependent effects on cysteine residues (Okamoto et al., 2001; Gu et al., 2002).

Though more than 20 MMP family members are expressed in humans, a select subset is expressed by resident cells of the central nervous system. These include MMP-2, which can be released from astrocytes and microglia, and MMP-9 which can be released from neurons and microglia (Conant et al., 2015). MMP-1 and MMP-3 are also released from neurons and glia and, along with MMP-2 and MMP-9, these family members have been implicated in structural and functional changes that enhance learning and memory (Meighan et al., 2006; Nagy et al., 2006; Almonte et al., 2013; Lonskaya et al., 2013; Wojtowicz and Mozrzymas, 2014; Allen et al., 2016; Wiera et al., 2017).

Matrix metalloproteases cleave a variety of substrates. Pathologically elevated levels of MMPs, or dysregulated localization of release, can contribute to neuronal injury (Asahi et al., 2000). Release occurring at physiologically appropriate levels, with localized proteolysis of specific substrates, is instead critical to neuroplasticity (Huntley, 2012; Sonderegger and Matsumoto-Miyai, 2014; Conant et al., 2015). Plasticity-relevant substrates include *trans*-synaptic adhesion molecules (Lonskaya et al., 2013), protease-activated G protein coupled receptors (Allen et al., 2016), and components of the perineuronal net (PNN) (Huntley, 2012; Sonderegger and Matsumoto-Miyai, 2014; Conant et al., 2015; Bozzelli et al., 2018).

### MMP Activity Contributes to Neuronal Arborization, Dendritic Spine Expansion, and Enhanced Learning and Memory

Several MMPs have been associated with enhanced dendritic arborization. For example, exogenous MMP-1 increases arborization of cultured murine hippocampal neurons and astrocyte-driven overexpression of human MMP-1 increases dendritic arborization in murine cortex as determined by Golgi staining and analysis (Allen et al., 2016). Conversely, MMP-2 deletion reduces dendritic arbor in cerebellar Purkinje cells, MMP-3 deletion reduces dendritic arbor in the visual cortex (Aerts et al., 2015; Verslegers et al., 2015), and MMP-9 deletion reduces both dendritic length and complexity in pyramidal neurons of the murine CA1 hippocampus (Murase et al., 2016). In related work, broad-spectrum MMP inhibitors have been shown to reduce neurite outgrowth in cultured cortical neurons (Sanz et al., 2015).

In terms of the potential to influence hippocampal function in a relatively rapid manner, MMPs have been shown to stimulate formation of spine head protrusions (Szepesi et al., 2013) and to increase the width or length of spines or their precursors (Tian et al., 2007; Wang et al., 2008; Stawarski et al., 2014). MMP-generated integrin binding ligands can also increase surface levels of dendritic GluA1 and can increase the frequency of mini excitatory post-synaptic currents, perhaps secondary to synaptic unsilencing (Lonskaya et al., 2013).

Consistent with their ability to quickly enhance synaptic plasticity, MMP-3 and -9 activity have been linked to long-term potentiation (LTP) of synaptic transmission in hippocampal regions (Nagy et al., 2006; Meighan et al., 2007; Conant et al., 2010; Wiera et al., 2017). LTP, one of the functional underpinnings of learning and memory, is reduced in models of depression (Riga et al., 2017) and its expression depends on the expansion and/or formation of dendritic spines. MMP-2 and -9 activity also enhance learning and memory in other regions including the striatum and the amygdala (Ganguly et al., 2013; Smith et al., 2014).

Though a full understanding of the mechanisms that underlie MMP-dependent neuroplasticity is not complete, several non-exclusive mechanisms have been described. These include generation of integrin binding ligands and integrin-dependent actin polymerization and spine expansion, cleavage-dependent activation of specific G protein coupled receptors, and activation of pro-neurotrophins (Lee et al., 2001; Conant et al., 2015). Integrin signaling, however, appears critical to MMP-dependent dendritic spine expansion (Nagy et al., 2006; Meighan et al., 2007; Wang et al., 2008; Lonskaya et al., 2013).

### MMP Activity May Contribute to Pyramidal Cell Disinhibition

An overall reduction in excitatory to inhibitory balance could follow from changes restricted to pyramidal cells. Emerging evidence, however, also supports a role for disinhibition of pyramidal cells as a potential mediator of antidepressant efficacy. Consistent with this, ketamine, which preferentially targets NMDA-responsive glutamate receptors on GABAergic interneurons (Widman and McMahon, 2018), can alleviate depression-related behavior in a rapid and sustained manner (Zarate et al., 2006). An allosteric modulator of a GABA A receptor subunit can similarly alleviate behavioral manifestations of depression (Zanos et al., 2017). Less directly, changes to perineuronal net (PNN) integrity might also promote pyramidal cell disinhibition (Frischknecht et al., 2009; Slaker et al., 2015; Favuzzi et al., 2017; Hayani et al., 2018). Several studies suggest that attenuation of the PNN may reduce PV interneuron activity, leading to pyramidal cell disinhibition. Without an intact PNN, glutamate may more easily diffuse from presynaptic release sites (Bikbaev et al., 2015). PNN disruption has also been linked to enhanced lateral diffusion of glutamate receptors on PV interneurons so that receptors are less well positioned to receive glutamate input (Frischknecht et al., 2009). Moreover, PNN disruption can increase membrane capacitance and thus reduce the ability of PV interneurons to fire (Tewari et al., 2018). Consistent with these studies, the frequency of mini excitatory post-synaptic potentials (mEPSCs) recorded from PV expressing fast spiking interneurons is reduced in mice that do not express brevican, an important PNN component (Favuzzi et al., 2017). In addition, PNN disruption observed 7 days after the administration of chondroitinase is associated with a reduction in both spontaneous and mini EPSCs recorded from PV interneurons (Hayani et al., 2018).

The potential for antidepressant medications to effect disinhibition is supported by rodent studies linking monoamine reuptake inhibitors to reductions in PNN integrity (Ohira et al., 2013; Guirado et al., 2014; Umemori et al., 2015). This could follow from the ability of monoamine reuptake inhibitors to increase expression of PNN-degrading proteases including MMP-9 (Tamasi et al., 2014; Murase et al., 2017; Wen et al., 2017). In a recent study, the serotonin and norepinephrine reuptake inhibitor venlafaxine increased hippocampal levels of MMP-9 protein and attenuated PNN expression (Alaiyed et al., 2019). In contrast, in a social defeat stress model, the depressive phenotype was associated with increased levels of PNN components (Riga et al., 2017). Work that examined the effects of social isolation, which also induces a depressive phenotype, demonstrated increases in PNNs in select regions of the amygdala and prefrontal cortex (Castillo-Gomez et al., 2017). In human work, changes in PNN staining have been noted with depression (Pantazopoulos and Berretta, 2016), and a polymorphism in the PNN constituent neurocan has been associated with increased risk of bipolar depression (Cichon et al., 2011).

Parvalbumin (PV)-expressing cells are the predominant GABA-releasing neuronal population that is surrounded by the PNN (Pizzorusso et al., 2002; Rossier et al., 2015; Lensjo et al., 2017a). Since each PV interneuron can contact hundreds of pyramidal cells (Andersen et al., 2006), PNN disruption may influence pyramidal cell activity at the population level. Consistent with this, recent findings in rodents show that PNN diminution has the potential to enhance both the power of gamma oscillations and the frequency of sharp wave ripple events (Lensjo et al., 2017b; Sun et al., 2018). Similarly, chronic venlafaxine treatment stimulates PNN diminution and an MMP-9-dependent increase in carbachol-induced gamma oscillatory power in *ex vivo* hippocampal slices (Alaiyed et al., 2019). While the activity of PV-expressing cells contributes to both sharp wave ripples and gamma oscillations, disinhibition of pyramidal cell activity can also influence these events. Pyramidal cell-derived sharp wave magnitude correlates with gamma power, and sharp waves initiate the sharp wave ripple complex (Sullivan et al., 2011). Importantly, gamma oscillations and sharp wave ripples are critical to memory encoding and consolidation (Buzsaki, 1989; Howard et al., 2003; Jutras and Buffalo, 2010), processes that may go awry in the setting of depression (MacQueen and Frodl, 2011).

Gamma oscillations associated with exercise and sharp wave ripples associated with sleep or quite restfulness are typically mutually exclusive, but the two rhythms are not fully independent. Recent work shows that exercise-induced enhancement of theta-nested gamma oscillations can stimulate a subsequent increase in sharp wave ripple area as detected with *in vivo* recordings (Zarnadze et al., 2016). Similarly, in *ex vivo* hippocampal slices, carbachol and kainate stimulated gamma oscillations are associated with a post-washout enhancement of sharp wave ripple area and event frequency (Zarnadze et al., 2016).

While future studies will be necessary to better elucidate the role of PNN modulation as a means to stimulate an increase in gamma oscillation power in humans, the potential significance of gamma to major depression is underscored in a recent review that focused on abnormalities in this rhythm as a potential biomarker of disease (Fitzgerald and Watson, 2018). Importantly, the power of gamma oscillations could represent a measurable correlate of monoamine and MMP-dependent enhancement of E/I balance.

## A Better Understanding of Antidepressant-Associated Increases in MMP Expression Could Lead to Novel Therapeutics

Currently available antidepressants have the potential to increase excitatory neurotransmission. Several lines of evidence link both enhanced excitatory transmission and glutamate receptor activation to increased MMP expression or release (Michaluk et al., 2007; Pauly et al., 2008; Conant et al., 2010; Wiera et al., 2012). In neurons, glutamate can stimulate increased DNA binding of AP-1 with subsequent rapid expression of MMP-9 (Bajor et al., 2012; Kuzniewska et al., 2013). In addition, pre-formed MMPs are observed in perisynaptic intra-vesicular stores (Sbai et al., 2008). Release of vesicular MMPs from non-neural cells is soluble NSF attachment protein receptor (SNARE) dependent (Kean et al., 2009); if a similar mechanism occurs in neurons and glia, MMP release might be facilitated by stimuli such as glutamate that can evoke increases in intracellular calcium.

Though the effects of monoamines on MMP release from brain cells have been less extensively studied, noradrenaline increases MMP-2 and MMP-9 activity in a hypothalamic slice preparation (Maolood et al., 2008). In striatal slice preparations (Li et al., 2016), dopamine and a D1 receptor agonist can upregulate MMP activity, as determined by an increase in MMP-dependent cleavage of β-dystroglycan. In non-neural cells, several studies suggest that monoamines can enhance MMP expression as well as MMP-dependent endpoints (Rietz and Spiers, 2012). For example, norepinephrine upregulates the expression of MMP-2 and MMP-9 in cultured nasopharyngeal carcinoma cells (Yang et al., 2006). Moreover, published work suggests that norepinephrine-induced remodeling of rat heart is MMP dependent (Briest et al., 2001).

Specific transcription factors may underlie monoamine dependent changes in MMP expression. We have observed that norepinephrine can increase DNA binding activity of AP-1 in brain-derived cells (Conant et al., 2004). CREB may also influence MMP expression (Xie et al., 1997), and this transcription factor is relevant to effects of monoaminergic antidepressants (Chen et al., 2001). β adrenergic receptors and 5-HT_7_ serotonin receptors are typically linked to Gαs signaling and therefore likely activate CREB (Shaywitz and Greenberg, 1999). Consistent with this are studies linking the 5-HT_7_ receptor to increased MMP-9 activity (Bijata et al., 2017). Additional studies are warranted to fully identify transcription factors that link the activation of specific monoamine receptor subtypes and enhanced MMP expression.

**FIGURE 1:**
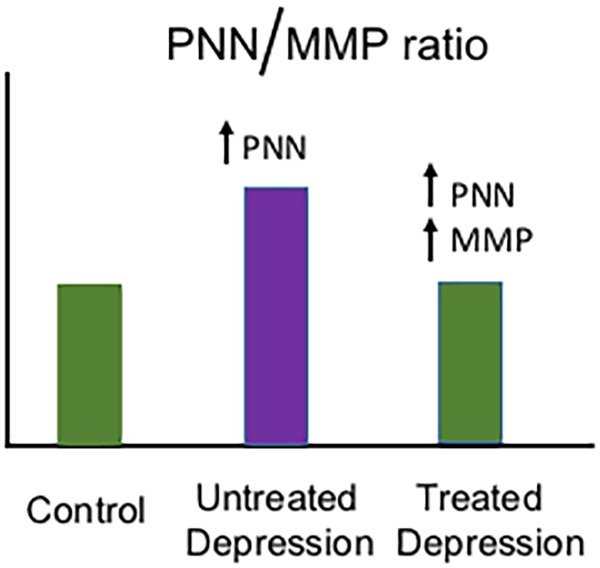
Hypothetical model of depression-related changes in the PNN/MMP ratio. Shown is a hypothetical histogram of potential changes in the PNN/MMP ratio (Y axis) in brain tissue from control patients, untreated but depressed patients, and depressed patients treated with a monoamine reuptake inhibitor (grouped along the X axis as indicated). Control patients have physiological PNN and MMP levels, while untreated patients with depression may have an increase in PNN deposition due to factors including stress (Riga et al., 2017) and thus an increase in the PNN/MMP ratio. In contrast, in depressed patients who are treated with monoamine reuptake inhibitors, MMP levels may increase and thus bring the PNN/MMP ratio back to control patient levels.

Monoamine modulators including the serotonin/norepinephrine reuptake inhibitor venlafaxine have been associated with an increase in MMP-9 mRNA expression (Tamasi et al., 2014) and MMP-9 protein levels (Alaiyed et al., 2019). Moreover, psychostimulants that increase monoamine levels including methamphetamine and cocaine have been associated with increased MMP-2- and MMP-9-dependent neuroplasticity (Mizoguchi et al., 2007, 2008; Conant et al., 2011; Smith et al., 2014). In Figure 1, we show a hypothetical schematic of PNN and MMP changes in the brains of normal individuals, those with untreated depression, and those with depression who are taking a monoamine reuptake inhibitor. While stress and the depressive phenotype have been associated with an increase in PNN deposition in a rat model (Riga et al., 2017), and imipramine can normalize PNN levels and concomitantly improve LTP (Riga et al., 2017), additional study will be necessary to determine whether depression and stress increase PNN levels in humans and whether antidepressant treatment can restore an adaptive PNN/MMP balance.

## MMP Modulation May Be Useful for Alzheimer’s Disease and Additional Disorders in Which Hippocampal Dysfunction is a Prominent Feature

It is critical to identify effective strategies to reduce the incidence of Alzheimer’s disease (AD), the most common form of dementia in individuals who are over age 65. Epidemiological studies suggest that education, social engagement, and exercise may reduce risk and/or delay the age of disease onset (Kivipelto et al., 2018). In contrast, untreated major depression can increase AD risk (Kivipelto et al., 2018).

Deficits in monoaminergic transmission have been implicated in the pathogenesis of Alzheimer’s disease (AD). For example, tau lesions within the locus coeruleus (LC), the predominant source of norepinephrine within the CNS, are identified early in disease, occurring up to 10 years prior to the onset of cognitive symptoms (Chalermpalanupap et al., 2013). Importantly, LC lesions increase Aβ plaque load and spatial memory deficits in a mouse model of AD (Kalinin et al., 2007). In related work, enhanced serotonergic signaling is linked to reduced Aβ deposition in human studies as well as in a murine model of AD (Sheline et al., 2014).

Importantly, both MMP-9 and the transmembrane spanning A Disintegrin and Metalloprotease-10 (ADAM-10) have been shown to act as α secretases (Jorissen et al., 2010; Fragkouli et al., 2012), which process amyloid precursor protein (APP) so that amyloid aggregates do not form. In a mouse model of AD, MMP-9 overexpression is neuroprotective (Fragkouli et al., 2014). In related work, overexpression of MMP-2, which shares considerable overlap in substrate specificity with MMP-9 but is typically less inducible, nearly abolished toxic amyloid-β production in HEK cells that overexpressed hAPP (Py et al., 2014). Furthermore, tissue inhibitor of matrix metalloprotease-3, which inhibits both ADAMs and soluble MMPs including MMP-9 (Brew and Nagase, 2010), increases amyloid aggregate- promoting β secretase-mediated processing of amyloid precursor protein (Hoe et al., 2007).

Based on work showing that conditional knockout of ADAM-10 in neural stem cells led to substantially reduced α secretase mediated processing of APP, it has been suggested that ADAM-10 represents the predominant brain-resident α secretase [(Jorissen et al., 2010); see also related work in Lammich et al. (1999), Skovronsky et al. (2000), Hartmann et al. (2002)]. While ADAM-10 may be important to protein kinase C-activated cleavage (Skovronsky et al., 2000), increases in inducible MMPs such as MMP-9 may upregulate α cleavage in select settings. This is supported by work showing α secretase activity is maintained in the majority of cell lines derived from embryonic ADAM-10 deficient fibroblasts (Hartmann et al., 2002). Intriguingly, ADAM-10 knockdown substantially reduces MMP-9 levels (Li et al., 2015; van der Vorst et al., 2015). Of additional interest, select agonists for the G protein coupled protease activated receptor-1, which include MMPs, can increase ADAM-10 activity (Li et al., 2014).

**FIGURE 2:**
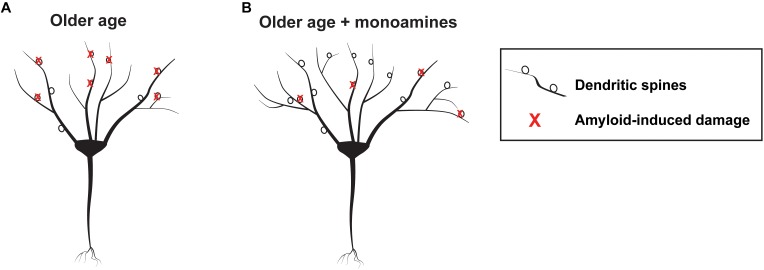
Schematic showing hypothetical monoamine dependent effects on the aging brain. At left is a schematic showing a representative neuron with amyloid mediated synaptic injury in older age **(A)** and a neuron having more arbor and synapses, as well as lesser amounts of peri-neuronal amyloid, in older age developing in the background of monoamine reuptake inhibition **(B)**.

Consistent with the potential of monoamines to enhance metalloprotease (MP) dependent APP processing, one group retrospectively compared brain amyloid load in elderly patients that were exposed to antidepressant drugs within the previous 5 years to control patients that were not (Sheline et al., 2014). Antidepressant-exposed individuals had substantially less amyloid load as quantified by positron emission tomography (PET) imaging. Intriguingly, cumulative time of antidepressant use within the period of interest correlated with a reduction in plaque load. Chronic treatment with the SSRI citalopram reduced plaque burden in mice, and related work suggested that serotonin could increase α secretase activity (Cirrito et al., 2011). Interestingly this effect is ERK-dependent, and ERK signaling has been linked to increased expression of both MMP-9 and ADAM-10 (Wan et al., 2012; Missan et al., 2015). Treatment of rodents with citalopram, however, did not increase expression of ADAM-10 or neprilysin, another α secretase (Cirrito et al., 2011). Importantly, in addition to their potential to enhance cognitive reserve and non-amyloidogenic APP processing as discussed previously, MMPs including MMP-2 can directly cleave amyloid (Hernandez-Guillamon et al., 2015) to reduce plaque load. An overview of mechanisms by which MMP activity could enhance cognitive reserve and reduce amyloid accumulation in individuals at risk for AD is diagrammed in Figure 2.

**FIGURE 3:**
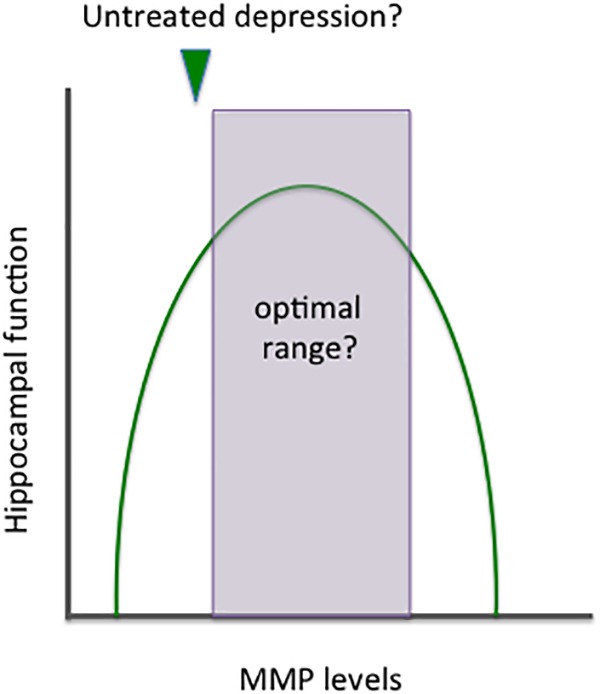
Benefits and risks of altering MMP expression. MMPs target a variety of CNS substrates and thus it is not surprising that their activity has been implicated in both neuroprotection and neuronal injury. Similar to norepinephrine, these enzymes appear to have an inverted U shaped curve with respect to benefit. Overall effects on plasticity likely depend not only on absolute levels but on MMP subtypes and cellular sources, as well as the location of their release. In depression, it may be of benefit to increase the expression of specific MMPs and/or shift overall levels to the right side of the area that remains within the inverted U shape.

Metalloproteinases target varied CNS substrates, and MMPs can thus enhance adaptive brain plasticity or stimulate inflammation and cytotoxicity. Though the *location* of release is also important, MMPs may have an inverted U shaped curve with respect to overall *levels* and benefit (see Figure 3).

## Summary and Future Directions

In summary, an appreciable body of evidence has emerged to suggest that major depressive disorder is linked to disrupted excitatory neurotransmission. This can follow from changes including reduced neuronal arborization and synapse formation as well as increased PNN deposition (Thompson S.M. et al., 2015; Riga et al., 2017). If untreated, these changes can predispose an individual to depression and/or an increased risk of dementia.

Future studies will be necessary to examine critical questions including whether MMPs contribute to behavioral improvement in depression or animal models, and whether potential changes to gamma oscillatory power or sharp wave ripple event frequency will be adaptive. With respect to this latter issue, it will be necessary to determine whether monoamine reuptake inhibitors and/or MMPs will lead to reactivation of learning-activated neuronal assemblies during sharp wave ripple events.

Finally, future studies should further explore the possibility that select antidepressants, alone or in or combination, will reduce or ameliorate Alzheimer’s pathology/symptomatology. This could follow from slowly evolving enhancement of cognitive reserve and reduced amyloid deposition. In addition, improved cognition in the short term could follow from PNN modulation with subsequently enhanced SWR frequency and/or gamma power (Insel et al., 2012; Iaccarino et al., 2016; Klein et al., 2016; Cattaud et al., 2018; Cowen et al., 2018; Stoiljkovic et al., 2018).

## Author Contributions

SA and KC wrote and revised the portions of this review.

## Conflict of Interest Statement

The authors declare that the research was conducted in the absence of any commercial or financial relationships that could be construed as a potential conflict of interest.
